# Distinct patterns of caries, erosive tooth wear, and hypomineralization in preschool children: a population-based study of associated factors

**DOI:** 10.1007/s00784-026-07179-x

**Published:** 2026-10-15

**Authors:** Jana Biermann, Angelika Puls, Trixie Heller, Lena Scholz, Lorenz Hein, Annette Wiegand

**Affiliations:** 1 Department of Preventive Dentistry, Periodontology and Cariology, University Medical Center Göttingen, Robert-Koch-Str. 40, Göttingen, 37075 Germany; 2 Public Health Department for the City and District of Göttingen, Theaterplatz 4, Göttingen, 37073 Germany

**Keywords:** Dental caries, Erosive tooth wear, Molar-incisor hypomineralization, Preschool children, Epidemiology, Risk factors

## Abstract

**Objectives:**

To assess prevalence, severity, and co-occurrence of dental caries, erosive tooth wear (ETW), and hypomineralization in preschool children, and to analyse their associations with general health, developmental, and sociodemographic factors.

**Materials and methods:**

This cross-sectional study was conducted within the mandatory school entry examination in Göttingen, Germany. A total of 1,461 children aged 5–6 years underwent standardized dental examinations assessing caries (dft/DFT), ETW (Basic Erosive Wear Examination), and hypomineralization (MIH Treatment Need Index). Information on general health, developmental, and sociodemographic factors was obtained from standardized parental questionnaires and medical assessments conducted as part of the school entry examination. Associations were analysed using multivariable regression models.

**Results:**

Caries experience was observed in 29.4% of children and showed a highly polarized distribution. ETW was present in 69.7% of children, generally with low severity. Hypomineralization affected 10.8% of children. No significant associations were found between the three conditions. Caries was strongly associated with migration background, low maternal education, developmental issues, and obesity. ETW showed limited associations, increasing with age and being slightly more severe in boys. Hypomineralization showed no consistent associations.

**Conclusions:**

These conditions were common but largely independent and showed differing patterns of association with sociodemographic and health-related factors.

**Clinical relevance:**

The largely independent occurrence of these oral conditions suggests that risk assessment and prevention should address them as distinct conditions with differing patterns of association.

**Supplementary Information:**

The online version contains supplementary material available at https://doi.org/10.1007/s00784-026-07179-x.

## Introduction

Oral health in early childhood is a key component of general health and well-being, with important implications for quality of life and long-term health trajectories [1]. Despite substantial improvements in oral health over recent decades, dental caries remains one of the most prevalent chronic diseases in children worldwide [1, 2]. Notably, the distribution of caries is highly uneven, with a large proportion of disease burden concentrated in a relatively small subgroup of affected individuals, reflecting persistent inequalities even in high-income countries [2, 3].

In addition to dental caries, other oral conditions such as erosive tooth wear (ETW) and molar-incisor hypomineralization (MIH) have gained increasing attention in pediatric populations. ETW is characterized by chemical loss of dental hard tissue, often influenced by mechanical factors [4] and has been reported to be prevalent even in young children, although typically of low severity [5–7]. Its etiology is multifactorial and includes dietary habits, particularly the frequent consumption of acidic foods and beverages, as well as possible systemic factors such as gastroesophageal reflux [7]. MIH is a qualitative developmental defect of enamel affecting at least one permanent first molar and often incisors [8]. It represents a clinically relevant condition due to hypersensitivity, increased caries susceptibility, and restorative challenges [9]. In addition to MIH affecting the permanent dentition, hypomineralization defects have also been described in the primary dentition, with potential links between both conditions [10]. Global prevalence estimates indicate that hypomineralization affects a considerable proportion of children, although reported rates vary widely [11, 12]. The etiology is considered multifactorial, with potential associations to prenatal, perinatal, and early childhood health conditions, yet remains incompletely understood [13, 14].

While these oral conditions are frequently investigated separately, comparatively little is known about their co-occurrence and potential interrelationships within the same population. Existing studies typically focus on single conditions or selected pairwise associations, and comprehensive analyses integrating multiple oral outcomes in early childhood remain scarce [15–18]. It therefore remains unclear whether these conditions share common determinants or occur independently. Moreover, population-based data in preschool children that simultaneously assess multiple oral conditions and link them to general health and developmental factors remain limited.

Therefore, this study aimed to assess the prevalence and severity of dental caries, ETW, and hypomineralization in a large population-based cohort of preschool children, to examine their co-occurrence, and to analyse their associations with general health, developmental, and sociodemographic factors.

## Materials and methods

### Study design and population

This cross-sectional study was conducted within the framework of the mandatory school entry examination in Göttingen, Germany. The school entry examination is a routine public health assessment performed prior to school enrolment and covers the vast majority of children of a given birth cohort, resulting in a study population with broad sociodemographic representation [19].

The study was carried out over one complete examination cycle. All children attending the main public health office for their school entry examination during the study period were invited to participate in an additional standardized dental examination as part of this study. Participation in the study was voluntary, and written informed consent was obtained from parents or legal guardians prior to examination.

A total of 1,678 children attended the school entry examination at the study site during the study period. Of these, 1,461 children underwent the dental examination and were included in the analysis, corresponding to a participation rate of approximately 87%.

Data collection was performed directly during the examination using a standardized protocol and recorded by trained staff.

The study was approved by the local ethics committee of the University Medical Center Göttingen (approval no.: 27/10/24) and conducted in accordance with the Declaration of Helsinki. The study was registered in the German Clinical Trials Register (DRKS-ID: DRKS00035446) and is reported in accordance with the STROBE guidelines [20].

### Oral examination

Dental examinations were performed by three trained and calibrated dentists under field conditions in rooms used for routine public health assessments, without the use of a dental treatment unit. Children were examined using magnification (SV2.7, starMed Germany; 2.7x), standardized light (starlight nano, starMed Germany), and dental mirrors. No radiographs or further diagnostic aids were used.

Calibration was conducted prior to and during the study. Inter-examiner reliability was assessed in a subsample of 86 children who were independently examined by pairs of examiners, covering all possible examiner combinations.

Agreement was evaluated using Cohen’s kappa and Gwet’s AC1 statistics and indicated excellent to perfect agreement across all indices, including caries (i)dmft/(I)DMFT, BEWE, and MIH-TNI (kappa values ranging from 0.961 to 1.00). Intra-examiner reliability was not assessed due to the study design, as repeated examinations were not feasible within the routine setting.

Due to the mixed dentition stage, either the primary or the permanent tooth was assessed at each tooth position, depending on which tooth was present. Teeth were only assessed when sufficiently erupted to allow reliable clinical evaluation.

Caries experience was recorded at the tooth level using the (i)dmft/(I)DMFT index, distinguishing between decayed (d/D), missing (m/M), and filled (f/F) teeth; initial carious lesions (i/I) were also recorded. To avoid misclassification of physiological tooth loss, the missing component (m/M) was excluded from the analyses. Caries experience at the child level was calculated as a combined dft/DFT score. This approach ensured that each tooth was counted only once in the absence of standardized methods for mixed dentition.

In addition, tooth-level analyses were performed separately for primary and permanent teeth.

ETW was assessed at the tooth level using the Basic Erosive Wear Examination (BEWE) [21], with scores from 0 (no ETW) to 3 (severe ETW). Sextant scores and cumulative BEWE sum scores were derived from the tooth-level assessments. Teeth that could not be reliably assessed due to extensive restorations or cavitated lesions were recorded as not assessable.

For child-level analyses, the presence of ETW was defined as BEWE ≥ 1.

Hypomineralization affecting both permanent and primary teeth was assessed at the tooth level using the MIH Treatment Need Index (MIH-TNI), which classifies affected teeth based on the presence of demarcated opacities, posteruptive enamel breakdown, and hypersensitivity [22]. For primary teeth, the same index was applied in accordance with the recent adaptation of the Würzburg concept, which considers the MIH-TNI classification applicable to both permanent and primary teeth without modification [23]. For analysis, hypomineralization was defined at the child-level as the presence of hypomineralization in at least one tooth, irrespective of dentition.

Fissure sealants were recorded dichotomously at the tooth level.

All findings were documented using a standardized electronic data entry system of the school entry examination, supplemented by an additional study-specific module developed for the present study. Data entry followed a standardized protocol and was performed directly during the examination. The examination and documentation procedures followed national recommendations for standardized oral health reporting in the German public health service [24].

### General health, developmental, and sociodemographic variables

Information on general health, developmental conditions, and sociodemographic characteristics was obtained as part of the routine school entry examination. Data collection was based on a standardized protocol and included parental questionnaires, structured interviews with parents conducted during the examination, and direct medical assessments performed by trained public health physicians.

The examination further incorporated objective measurements (e.g., height, weight, vision and hearing screening, and motor assessments) as well as a review of medical records, including child health booklets.

Variables were grouped into clinically meaningful categories to reflect distinct domains of child health and to reduce model complexity. Four groups were defined:

Perinatal factors, including preterm birth and low birth weight; developmental conditions, including language problems, delayed walking, early intervention, and neurological conditions; chronic conditions, including ear, nose, and throat conditions, respiratory, cardiovascular, and urinary tract conditions; and psychosocial and behavioural conditions, including psychosocial problems, behavioural difficulties, and disabilities.

Each group variable was coded dichotomously (0 = no condition, 1 = at least one condition within the respective group).

Rare conditions (e.g., measles, mumps, pertussis, chickenpox) were excluded from regression analyses due to extremely low prevalence and resulting unstable model estimates.

Detailed definitions of all variables and grouping procedures are provided in the supplementary material (Table S1).

### Statistical analysis

All statistical analyses were performed using R (R Foundation for Statistical Computing, Vienna, Austria, version 2026.01.1 + 403).

Descriptive statistics were calculated for all variables. Categorical variables are presented as absolute and relative frequencies, while continuous variables are reported as means with standard deviations.

Group comparisons were performed using chi-square or Fisher’s exact tests for categorical variables and Wilcoxon rank-sum tests for continuous variables, as appropriate.

To characterize the distribution of caries experience, the Significant Caries Index (SiC) [25] and the Specific Affected Caries Index (SaC) [26] were calculated.

Associations between oral health outcomes and general health as well as sociodemographic factors were analysed using multivariable regression models. Binary outcomes (prevalence of caries experience, ETW, and hypomineralization) were analysed using logistic regression models, and results are presented as odds ratios (OR) with 95% confidence intervals (CI).

For count outcomes (dft/DFT), Poisson and negative binomial regression models were initially fitted. Due to substantial overdispersion (variance-to-mean ratio ≈ 5.3; dispersion test *p* < 0.001), negative binomial regression was selected as the primary modelling approach, supported by improved model fit (lower AIC).

The severity of ETW (BEWE sum score) was analysed using linear regression models. Model assumptions were assessed using residual plots (residuals vs. fitted values and Q–Q plots). Minor deviations from normality were observed but were considered acceptable given the large sample size.

Fully adjusted models including all health group variables (perinatal risk factors, developmental conditions, chronic conditions, and psychosocial and behavioural conditions) and sociodemographic confounders (age, sex, migration background, number of siblings, parental education, and BMI category) constituted the primary analyses.

Potential multicollinearity between health variables was assessed using correlation analysis (phi coefficients for binary variables). Correlations were generally low to moderate, and no relevant multicollinearity was assumed.

Complete-case analysis was performed for all regression models. Observations with missing values in any of the variables included in a given model were excluded. In fully adjusted models, 1,091 children were included in the analysis.

A significance level of *p* < 0.05 was considered statistically significant.

## Results

### Study population

A total of 1,461 children (51.8% boys, 48.2% girls) were included in the analysis. The study population mainly consisted of children aged 5 and 6 years, with only a very small proportion of younger or older children. 47% of children had at least one erupted permanent tooth at the time of examination, consistent with the transitional stage of the mixed dentition. Among these children, the median number of permanent teeth was 3, and the majority had at least one erupted first permanent molar.

### Oral health outcomes

Dental caries experience (dft/DFT > 0) was observed in 29.4% of children. The distribution was highly skewed, with a pronounced polarization of disease burden, as reflected by a high Significant Caries Index (SiC = 3.9) and Specific Affected Caries Index (SaC = 4.4), and with 20% of children accounting for the majority of the total caries burden.

ETW (BEWE ≥ 1) was present in 69.7% of children, with generally low severity.

Hypomineralization affected 10.8% of children overall. Detailed results on prevalence, severity, and child-level distribution are presented in Table 1. Tooth-level distributions of oral conditions in primary and permanent teeth are presented in Tables 2 and 3.

**Table 1:** Oral health outcomes of the study population (N = 1,461)

**Caries experience **(permanent and primary dentition combined)			
	Prevalence		
		dft/DFT > 0, n (%)	429 (29.4)
		idft/IDFT > 0, n (%)	528 (36.1)
	Severity		
		dft/DFT, mean ± SD	1.29 ± 2.62
		idft/IDFT, mean ± SD	1.60 ± 2.92
	Polarization		
		Significant Caries Index (SiC), mean	3.87
		Specific Affected Caries Index (SaC), mean	4.39
	Treatment status		
		Untreated caries (dt/DT > 0), n (%)	268 (18.3)
		Restoration rate, % *	52.3
**Erosive tooth wear **(permanent and primary dentition combined)			
	Prevalence (BEWE ≥ 1), n (%)		1019 (69.7)
	Severity (BEWE sum score), (mean ± SD)		2.09 ± 2.10
	Number of affected teeth in children with BEWE ≥ 1, (mean ± SD)		2.18 ± 2.15
	BEWE Risk category [21]		
		None (BEWE sum score ≤ 2), n (%)	937 (64.1)
		Low (BEWE sum score 3–8), n (%)	505 (34.6)
		Medium (BEWE sum score 9–13), n (%)	19 (1.3)
		High (BEWE sum score ≥ 14), n (%)	none
**Hypomineralization**			
	Prevalence		
		Permanent dentition, n (%)	94 (6.4)
		Primary dentition, n (%)	79 (5.4)
		Combined, n (%)	158 (10.8)

* Restoration rate calculated as filled teeth/(decayed teeth + filled teeth).

**Table 2:** Tooth-level distribution of oral conditions in the primary dentition

			Caries experience			ETW (BEWE)					Hypomineralization (MIH-TNI)								
			n (%)			n (%)					n (%)								
Tooth	n (present)	missing, n	d	f	i	0	1	2	3	NA	0	1	2a	2b	2c	3	4a	4b,4c	NA
**55**	1453	6	65 (4.5)	90 (6.2)	56 (3.9)	1367 (94.1)	26 (1.8)	6 (0.4)	0	54 (3.7)	1341 (92.3)	54 (3.7)	3 (0.2)	0	0	1 (0.1)	0	0	54 (3.7)
**54**	1429	32	69 (4.8)	89 (6.2)	15 (1.0)	995 (69.6)	321 (22.5)	53 (3.7)	4 (0.3)	56 (3.9)	1367 (95.7)	6 (0.4)	0	0	0	0	0	0	56 (3.9)
**53**	1456	5	17 (1.2)	13 (0.9)	11 (0.8)	1413 (97.0)	28 (1.9)	13 (0.9)	0	2 (0.1)	1454 (99.9)	0	0	0	0	0	0	0	2 (0.1)
**52**	1395	62	42 (3.0)	12 (0.9)	13 (0.9)	1372 (98.4)	9 (0.6)	6 (0.4)	3 (0.2)	5 (0.4)	1391 (99.7)	0	0	0	0	0	0	0	4 (0.3)
**51**	1214	179	58 (4.8)	28 (2.3)	15 (1.2)	1186 (97.7)	16 (1.3)	4 (0.3)	3 (0.2)	5 (0.4)	1208 (99.5)	0	1 (0.1)	0	0	0	0	0	5 (0.4)
**61**	1212	175	63 (5.2)	19 (1.6)	13 (1.1)	1183 (97.6)	15 (1.2)	6 (0.5)	2 (0.2)	6 (0.5)	1205 (99.4)	0	1 (0.1)	0	0	0	0	0	6 (0.5)
**62**	1393	67	40 (2.9)	7 (0.5)	16 (1.1)	1375 (98.7)	7 (0.5)	8 (0.6)	1 (0.1)	2 (0.1)	1391 (99.9)	0	0	0	0	0	0	0	2 (0.1)
**63**	1450	8	16 (1.1)	10 (0.7)	5 (0.3)	1406 (97.0)	31 (2.1)	11 (0.8)	1 (0.1)	1 (0.1)	1449 (99.9)	0	0	0	0	0	0	0	1 (0.1)
**64**	1429	32	70 (4.9)	103 (7.2)	14 (1.0)	822 (57.5)	494 (34.6)	48 (3.4)	7 (0.5)	58 (4.1)	1369 (95.8)	1 (0.1)	1 (0.1)	0	0	0	0	0	58 (4.1)
**65**	1451	9	75 (5.2)	94 (6.5)	35 (2.4)	1346 (92.8)	48 (3.3)	6 (0.4)	2 (0.1)	49 (3.4)	1351 (93.1)	45 (3.1)	1 (0.1)	3 (0.2)	1 (0.1)	0	1 (0.1)	0	49 (3.4)
**75**	1435	22	92 (6.4)	117 (8.2)	73 (5.1)	1102 (76.8)	254 (17.7)	20 (1.4)	1 (0.1)	58 (4.0)	1349 (94.0)	20 (1.4)	5 (0.3)	1 (0.1)	0	1 (0.1)	1 (0.1)	0	58 (4.0)
**74**	1431	29	93 (6.5)	126 (8.8)	30 (2.1)	616 (43.0)	587 (41.0)	146 (10.2)	8 (0.6)	74 (5.2)	1356 (94.8)	1 (0.1)	0	0	0	0	0	0	74 (5.2)
**73**	1460	1	4 (0.3)	6 (0.4)	7 (0.5)	1455 (99.7)	2 (0.1)	3 (0.2)	0	0	1459 (99.9)	0	0	0	0	1 (0.1)	0	0	0
**72**	1320	97	2 (0.2)	0	2 (0.2)	1319 (99.9)	1 (0.1)	0	0	0	1320 (100)	0	0	0	0	0	0	0	0
**71**	812	179	2 (0.2)	0	2 (0.2)	811 (99.9)	1 (0.1)	0	0	0	812 (100)	0	0	0	0	0	0	0	0
**81**	793	189	4 (0.5)	0	1 (0.1)	792 (99.9)	1 (0.1)	0	0	0	793 (100)	0	0	0	0	0	0	0	0
**82**	1309	101	4 (0.3)	0	2 (0.2)	1307 (99.8)	1 (0.1)	1 (0.1)	0	0	1309 (100)	0	0	0	0	0	0	0	0
**83**	1455	2	7 (0.5)	7 (0.5)	5 (0.3)	1449 (99.6)	4 (0.3)	1 (0.1)	0	1 (0.1)	1453 (99.9)	0	0	0	0	1 (0.1)	0	0	1 (0.1)
**84**	1428	32	92 (6.4)	130 (9.1)	29 (2.0)	667 (46.7)	541 (37.9)	140 (9.8)	10 (0.7)	70 (4.9)	1355 (94.9)	3 (0.2)	0	0	0	0	0	0	70 (4.9)
**85**	1439	18	76 (5.3)	121 (8.4)	78 (5.4)	1107 (76.9)	255 (17.7)	16 (1.1)	2 (0.1)	59 (4.1)	1352 (94.0)	21 (1.5)	3 (0.2)	2 (0.1)	0	0	2 (0.1)	0	59 (4.1)

n (present): number of present teeth, missing: number of missing teeth, d = decayed (cavitated lesion) tooth, f = filled tooth, i = tooth with initial (non-cavitated) lesion, NA = non-assessable tooth (e.g. due to extensive restorations or severe destruction). MIH-TNI categories 4b and 4c were combined for presentation due to the absence of observations in both categories

**Table 3:** Tooth-level distribution of oral conditions in the permanent dentition

			Caries experience			ETW (BEWE)					MIH (MIH-TNI)						
			*n* (%)			*n* (%)					*n* (%)						
Tooth	*n* (present)	missing, *n*	D	F	I	0	1	2	3	NA	0	1	2a	2b, 2c	3	4a-c	NA
**16**	349	1112	1 (0.3)	3 (0.9)	5 (1.4)	348 (99.7)	1 (0.3)	0	0	0	292 (83.7)	55 (15.8)	1 (0.3)	0	1 (0.3)	0	0
**15**	3	0	1 (33.3)	1 (33.3)	0	3 (100)	0	0	0	0	3 (100)	0	0	0	0	0	0
**12**	4	0	0	0	0	4 (100)	0	0	0	0	4 (100)	0	0	0	0	0	0
**11**	69	0	0	0	0	68 (98.6)	1 (1.4)	0	0	0	65 (94.2)	3 (4.3)	0	0	1 (1.4)	0	0
**21**	74	0	1 (1.4)	0	1 (1.4)	74 (100)	0	0	0	0	71 (95.9)	3 (4.1)	0	0	0	0	0
**22**	1	0	0	0	0	1 (100)	0	0	0	0	1 (100)	0	0	0	0	0	0
**23**	3	0	0	0	1 (33.3)	1 (33.3)	1 (33.3)	1 (33.3)	0	0	3 (100)	0	0	0	0	0	0
**25**	1	0	0	1 (100)	0	0	0	0	0	1 (100)	0	0	0	0	0	0	1 (100)
**26**	360	1101	1 (0.3)	1 (0.3)	4 (1.1)	360 (100)	0	0	0	0	310 (86.1)	47 (13.1)	2 (0.6)	0	1 (0.3)	0	0
**36**	349	1112	1 (0.3)	2 (0.6)	10 (2.9)	348 (99.7)	1 (0.3)	0	0	0	318 (91.1)	30 (8.6)	0	0	1 (0.3)	0	0
**35**	4	0	1 (25.0)	0	0	3 (75.0)	1 (25.0)	0	0	0	4 (100)	0	0	0	0	0	0
**34**	1	0	0	1 (100)	0	1 (100)	0	0	0	0	1 (100)	0	0	0	0	0	0
**32**	44	0	0	0	0	44 (100)	0	0	0	0	43 (97.7)	1 (2.3)	0	0	0	0	0
**31**	469	0	0	0	0	469 (100)	0	0	0	0	456 (97.2)	13 (2.8)	0	0	0	0	0
**41**	479	0	0	0	0	479 (100)	0	0	0	0	468 (97.7)	11 (2.3)	0	0	0	0	0
**42**	51	0	0	0	1	51 (100)	0	0	0	0	51 (100)	0	0	0	0	0	0
**43**	4	0	0	0	0	4 (100)	0	0	0	0	4 (100)	0	0	0	0	0	0
**44**	1	0	0	0	0	0	1 (100)	0	0	0	1 (100)	0	0	0	0	0	0
**45**	4	0	2 (50.0)	0	0	3 (75.0)	1 (25.0)	0	0	0	4 (100)	0	0	0	0	0	0
**46**	333	1128	0	4 (1.2)	9 (2.7)	333 (100)	0	0	0	0	297 (89.2)	35 (10.5)	0	0	1 (0.3)	0	0

n (present): number of present teeth, missing: number of missing teeth, D = decayed (cavitated lesion) tooth, F = filled tooth, I = tooth with initial (non-cavitated) lesion. NA = non-assessable tooth (e.g. due to extensive restorations or severe carious destruction). MIH-TNI categories 2b and 2c, as well as 4a–c, were combined for presentation due to the absence of observations. Permanent teeth with no erupted instances in the study population (n = 0) were excluded from the table

Among children with at least one erupted permanent first molar, 10.1% (95% CI: 7.8–13.1) had at least one fissure sealant. On the tooth level, 8.8% of erupted first permanent molars were sealed.

No significant associations were observed between dental caries experience, ETW, and hypomineralization (all *p* > 0.05).

No sex differences were observed for the prevalence of dental caries experience or hypomineralization. For ETW, prevalence did not differ by sex; however, boys showed slightly higher BEWE sum scores compared to girls (*p* = 0.01, Table 4).

**Table 4:** Sex differences in oral health outcomes

Outcome			Boys (*N* = 757)	Girls (*N* = 704)	*p*-value
**Caries experience**					
	Prevalence (dft/DFT > 0), n (%)		225 (29.7)	204 (29.0)	0.74
	Severity (dft/DFT), mean ± SD		1.29 ± 2.54	1.29 ± 2.7	0.75
	Untreated caries (dt/DT > 0), n (%)		143 (18.9)	125 (17.8)	0.58
	Restoration rate, % *		49.9	54.8	0.31
**Erosive tooth wear**					
	Prevalence (BEWE ≥ 1), n (%)		536 (70.8)	483 (68.6)	0.36
	Severity (BEWE sum score), mean ± SD		2.27 ± 2.27	1.9 ± 1.88	**0.01**
**Hypomineralization**					
	Prevalence, n (%)		77 (10.2)	81 (11.5)	0.41

* Restoration rate calculated as filled teeth/(decayed teeth + filled teeth)

### Associations with general health, developmental and sociodemographic factors

General health, sociodemographic background and developmental characteristics of the study population are summarized in Table 5.

**Table 5:** General health, sociodemographic background and developmental characteristics of the study population

Variable		Category	n (%)
**Sociodemographic and household characteristics**			
	Age	< 6 years	723 (49.5)
		6 years	721 (49.3)
		7 years	17 (1.2)
	Sex	male	757 (51.8)
		female	704 (48.2)
	Migration background	none	786 (61.7)
		one parent foreign-born	186 (14.6)
		both parents foreign-born	302 (23.7)
	Parental education	Mother with vocational training	1079 (86.7)
		Mother without vocational training	166 (13.3)
		Father with vocational training	1052 (90.1)
		Father without vocational training	116 (9.9)
	Number of siblings	0	272 (19.0)
		1	698 (48.6)
		≥ 2	465 (32.4)
**Developmental conditions**			
	Language problems	yes	730 (50.6)
		no	712 (49.4)
	Delayed walking	yes	22 (1.6)
		no	1384 (98.4)
	Early intervention	yes	366 (25.8)
		no	1051 (74.2)
	Neurological conditions	yes	728 (50.6)
		no	712 (49.4)
**Perinatal factors**			
	Preterm birth	yes	116 (7.9)
		no	1345 (92.1)
	Low birthweight	yes	104 (7.1)
		no	1357 (92.9)
**Chronic health conditions**			
	Ear, nose, and throat conditions	yes	351 (25.1)
		no	1047 (74.9)
	Respiratory conditions	yes	55 (3.8)
		no	1390 (96.2)
	Cardiovascular conditions	yes	51 (3.5)
		no	1395 (96.5)
	Urinary tract conditions	yes	80 (5.7)
		no	1318 (94.3)
**Psychosocial and behavioural conditions**			
	Psychosocial conditions	yes	160 (11)
		no	1298 (89)
	Behavioural conditions	yes	1008 (69.1)
		no	450 (30.9)
	Disability	yes	110 (7.6)
		no	1343 (92.4)
**History of infectious diseases**			
	Measles	yes	4 (0.3)
		no	1394 (99.7)
	Mumps	yes	2 (0.1)
		no	1395 (99.9)
	Varicella	yes	38 (2.7)
		no	1360 (97.3)
	Pertussis	yes	11 (0.8)
		no	1387 (99.2)
	**Other health indicators**		
	Body Mass Index (BMI) category	underweight	40 (2.8)
		normal weight	1259 (86.9)
		overweight	91 (6.3)
		obesity	59 (4.1)
	Completion of routine child health check-ups	complete	911 (68.9)
		incomplete	411 (31.1)

Percentages are based on available data.

#### Caries experience

Developmental conditions were associated with higher odds of caries experience (OR = 1.6, 95% CI: 1.1–2.4), whereas perinatal risk factors were associated with lower odds (OR = 0.6, 95% CI: 0.3–1.0).

Migration background was strongly related to caries experience, with children with two migrated parents showing markedly higher odds (OR = 4.0, 95% CI: 2.7–5.8).

Low maternal education was also associated with higher odds of caries experience (OR = 2.9, 95% CI: 1.8–4.9) and higher dft/DFT values (IRR = 1.81, 95% CI: 1.1–3.1).

In addition, obesity was strongly associated with both caries experience (OR = 4.5, 95% CI: 2.1–10.2) and severity (IRR = 3.2, 95% CI: 1.5–7.8).

A small inverse association was observed for chronic conditions in the count model.

No other variables were significantly associated with caries experience.

#### Erosive tooth wear

The number of siblings was associated with ETW, with children having one sibling showing lower odds compared to single children (OR = 0.7, 95% CI: 0.5–1.0).

In the analysis of BEWE sum scores, perinatal risk was associated with higher scores (β = 0.4, 95% CI: 0.0–0.8). Higher scores were also observed in children of mothers without vocational training (β = 0.5, 95% CI: 0.00–0.9), increased with age (β = 2.2, 95% CI: 0.8–3.6), and were slightly higher in boys than in girls (β = 0.3, 95% CI: 0.1–0.5).

No other variables were significantly associated with ETW.

#### Hypomineralization

The number of siblings was associated with hypomineralization, with higher odds in children with siblings compared to single children (OR = 2.2, 95% CI: 1.2–4.5). No other variables were significantly associated.

Full regression results are presented in Table 6.

**Table 6:** Associations between general health factors and oral health outcomes. OR = odds ratio; IRR = incidence rate ratio; β = regression coefficien

Predictor	Caries experience				ETW				Hypomineralization	
	Prevalence		Severity		Prevalence		Severity		Prevalence	
	OR (95% CI)	*p*-value	IRR (95% CI)	*p*-value	OR (95% CI)	*p*-value	β (95% CI)	*p*-value	OR (95% CI)	*p*-value
Developmental conditions (yes)	1.63 (1.11–2.42)	0.014	1.48 (1.02–2.13)	0.036	0.87 (0.64–1.17)	0.351	−0.15 (−0.42-0.12)	0.265	0.76 (0.50–1.17)	0.215
Perinatal factors (yes)	0.55 (0.29–0.98)	0.049	0.78 (0.45–1.39)	0.360	1.63 (1.01–2.73)	0.051	0.42 (0.01–0.82)	0.043	0.88 (0.41–1.70)	0.724
Chronic conditions (yes)	0.84 (0.59–1.19)	0.327	0.71 (0.50–1.00)	0.044	0.79 (0.60–1.05)	0.104	−0.12 (−0.37-0.14)	0.373	0.81 (0.53–1.24)	0.344
Psychosocial and behavioral conditions (yes)	1.07 (0.75–1.51)	0.697	1.32 (0.94–1.87)	0.109	0.80 (0.59–1.07)	0.132	0.00 (−0.27-0.27)	0.979	0.90 (0.56–1.41)	0.649
Age (years)	1.24 (0.25–6.22)	0.782	1.48 (0.36–11.53)	0.635	1.08 (0.24–7.60)	0.924	2.23 (0.83–3.64)	0.002	3.29 (0.44–16.76)	0.180
Sex (male vs. female)	1.14 (0.83–1.57)	0.422	0.99 (0.72–1.37)	0.968	1.09 (0.83–1.43)	0.528	0.29 (0.05–0.53)	0.019	0.94 (0.63–1.40)	0.757
One parent foreign born	1.63 (1.03–2.52)	0.032	1.83 (1.17–2.90)	0.008	0.96 (0.66–1.43)	0.851	0.09 (−0.25-0.44)	0.595	0.71 (0.36–1.28)	0.278
Both parents foreign born	3.97 (2.74–5.76)	<0.001	4.28 (2.92–6.34)	<0.001	0.89 (0.63–1.28)	0.539	−0.30 (−0.62-0.03)	0.071	0.76 (0.43–1.29)	0.327
Number of siblings: 1	0.90 (0.59–1.41)	0.647	0.74 (0.48–1.13)	0.164	0.68 (0.47–0.99)	0.049	−0.12 (−0.45-0.20)	0.458	2.19 (1.17–4.48)	0.021
Number of siblings: ≥2	1.10 (0.69–1.75)	0.695	0.98 (0.62–1.55)	0.942	0.80 (0.53–1.20)	0.291	0.06 (−0.30-0.41)	0.762	2.24 (1.14–4.72)	0.025
Mother without vocational training	2.92 (1.75–4.87)	<0.001	1.81 (1.09–3.05)	0.032	1.60 (0.94–2.77)	0.089	0.46 (0.00–0.92)	0.050	0.51 (0.19–1.22)	0.158
Father without vocational training	1.61 (0.92–2.79)	0.092	1.74 (1.02–3.02)	0.059	0.77 (0.45–1.34)	0.350	0.21 (−0.27-0.70)	0.388	0.90 (0.33–2.13)	0.817
Underweight	1.35 (0.49–3.43)	0.540	1.77 (0.73–5.05)	0.239	1.51 (0.60–4.61)	0.418	−0.17 (−0.61-0.95)	0.670	1.59 (0.45–4.43)	0.414
Overweight	1.05 (0.55–1.94)	0.872	0.91 (0.49–1.82)	0.787	1.29 (0.73–2.41)	0.399	0.43 (−0.09-0.95)	0.105	1.32 (0.53–2.86)	0.516
Obesity	4.53 (2.07–10.19)	<0.001	3.21 (1.51–7.83)	0.003	1.13 (0.54–2.54)	0.762	−0.04 (−0.72-0.65)	0.917	1.67 (0.53–4.31)	0.331

Reference categories: female sex, no migration background, no siblings, parental vocational training, normal weight, and absence of the respective condition.

## Discussion

This study provides a comprehensive assessment of multiple oral health conditions in a large, population-based cohort of preschool children. Dental caries, ETW, and hypomineralization were common but showed no consistent associations with each other. Sociodemographic factors were strongly associated with caries, whereas associations with general health and developmental variables differed across conditions.

The prevalence of caries experience observed in this study is in line with previous reports from high-income countries [2] and national data from Germany [3, 27]. A pronounced polarization was evident, with a substantial proportion of the disease burden concentrated in a relatively small subgroup of children. This pattern has been widely described in both national and global data, reflecting the socially patterned distribution of oral diseases [3, 28, 29]. The degree of polarization observed in the present study appeared particularly marked, which may partly reflect the study setting within a school entry examination framework, that facilitates the inclusion of children from diverse sociodemographic backgrounds compared to studies relying solely on voluntary participation.

The prevalence of ETW observed in this study was high, while overall severity remained low. This is consistent with findings from European studies in similar age groups [5, 6, 30]. However, the prevalence was higher than estimates reported in systematic reviews [7, 31]. These differences may reflect temporal trends, as well as variations in study design and population characteristics. The combination of high prevalence and low severity may also indicate that mild erosive changes in primary teeth partly reflect physiological processes rather than clinically relevant disease [5, 32]. Compared to previous findings from the same region [5], the prevalence observed in the present study was slightly lower. This difference may be explained by variations in study setting and sampling, as earlier analyses were conducted in kindergarten-based cohorts, whereas the present study was embedded in a population-based school entry examination with broader coverage.

The prevalence of MIH was lower than reported in previous studies [11, 18], likely due to the relatively young age of the study population, as not all permanent teeth relevant for diagnosis had erupted at the time of examination. Consequently, some cases may have remained undetected, potentially leading to an underestimation of the true prevalence.

No consistent associations were observed between dental caries experience, ETW, and hypomineralization. While previous studies have reported associations between specific conditions, particularly between MIH and caries [15, 18], evidence from studies assessing multiple oral conditions simultaneously remain limited [16, 17]. The lack of consistent associations suggests that these conditions may occur largely independently in early childhood. However, smaller subgroup sizes may have limited the ability to detect weak associations.

Sociodemographic factors, particularly migration background and parental education, showed strong and consistent associations with caries experience. These findings are in line with previous research highlighting the importance of social determinants in oral health [29] and reflect patterns observed in both national data [3] and global reports [1]. The clear gradient observed for migration background suggests that social and structural factors continue to influence caries distribution already in early childhood. In addition, obesity was strongly associated with both caries experience and severity in the present study. This finding is consistent with previous evidence suggesting a positive association between higher body mass index and dental caries in children, likely reflecting shared behavioral risk factors such as dietary patterns characterized by high sugar intake [33]. However, given the cross-sectional design and the relatively wide confidence intervals, this association should be interpreted carefully.

Beyond sociodemographic influences, developmental conditions were associated with higher odds of caries experience and increased caries severity. This may reflect challenges associated with developmental delays, potentially affecting oral hygiene practices, dietary habits, or access to preventive care. These findings are consistent with previous findings showing that developmental vulnerability at school entry is associated with unmet dental needs and barriers to care [34].

In contrast, inverse associations were observed for perinatal risk factors and chronic conditions. These findings should be interpreted with caution and may reflect differences in healthcare utilization, as children with medical conditions may have more frequent contact with healthcare services and receive increased preventive attention. Previous studies have reported inconsistent associations between perinatal factors and caries [35].

For ETW, no consistent associations with general health variables were observed. Instead, age emerged as a key determinant, reflecting the cumulative nature of the condition. Slightly higher BEWE scores in boys and in children of mothers without vocational training suggest that lifestyle-related and social factors may play a role, although less consistently than for caries. These findings are in line with previous studies describing ETW as primarily influenced by age, dietary habits, and other behavioural factors [7, 36]. The observed association with perinatal risk factors should be interpreted cautiously. While direct evidence linking perinatal conditions to ETW is limited, potential pathways may include differences in early feeding practices, increased exposure to medications, or a higher prevalence of gastroesophageal reflux in children born preterm or with low birth weight. These factors have been discussed as potential contributors to ETW in early childhood [7, 36].

Hypomineralization showed no consistent associations with general health or sociodemographic variables, which is in line with its multifactorial and not yet fully understood etiology [8, 13]. The observed association with the number of siblings may reflect shared environmental exposures but should be interpreted with caution.

Taken together, these findings indicate that different oral conditions in early childhood are associated with distinct patterns of sociodemographic and health-related factors. While social inequalities appear to play a major role in the distribution of caries, additional factors contribute to variation across conditions, highlighting the need for differentiated prevention strategies.

Strengths of this study include the large sample size and the population-based design within a mandatory school entry examination, allowing for broad coverage of the target population.

In addition, multiple oral health outcomes were assessed simultaneously using standardized indices, and linked to detailed general health, developmental, and sociodemographic data.

Several limitations should be considered. Due to the cross-sectional design, causal relationships cannot be inferred. Examinations were conducted under field conditions without dental treatment units or radiographs, which may have limited the detection of early lesions.

Caries experience was assessed using a modified dft/DFT index excluding the missing component (m/M) in order to avoid misclassification of physiological tooth loss in the mixed dentition. Although most missing primary teeth were anterior teeth and therefore consistent with physiological exfoliation, 180 missing primary molars were recorded, affecting 98 children. Physiological exfoliation of primary molars generally occurs between 9 and 12 years of age [37], well beyond the age of the children included in the present study. As primary molars are among the primary teeth most frequently affected by dental caries [38], at least some of the missing primary molars were likely extracted because of severe caries. Consequently, excluding the missing component may have resulted in a slight underestimation of caries experience. Direct comparisons with studies using the conventional dmft/DMFT index should therefore be interpreted with caution.

Analyses were performed at the child level across dentitions, potentially obscuring dentition-specific associations. However, stratified analyses would have substantially reduced sample size and statistical power and were therefore not considered feasible within the present study design. Furthermore, the study population was drawn from a single urban region, which may limit the generalizability of the findings to children living in rural areas or other settings. In addition, detailed information on behavioural factors such as oral hygiene practices, dietary habits, or fluoride exposure was not available, as data collection was restricted to variables routinely assessed within the school entry examination. These factors may have contributed to residual confounding. Finally, grouping of general health variables may have masked more specific associations, and complete-case analysis may have introduced some degree of selection bias.

Overall, these findings emphasize the importance of considering multiple oral conditions and their differing determinants when designing preventive strategies in early childhood.

## Supplementary Information

Below is the link to the electronic supplementary material.

Supplementary Material 1

## Data Availability

The datasets generated and analysed during the current study are not publicly available due to legal and data protection restrictions concerning sensitive health data of minors.
