# Supplementary material for: Distinct patterns of caries, erosive tooth wear, and hypomineralization in preschool children: a population-based study of associated factors
Source: Clin Oral Investig. 2026 Oct 15;30(11):530. doi: 10.1007/s00784-026-07179-x (PMC13672554; doi:10.1007/s00784-026-07179-x)
Supplement: Supplementary file 1 [file 784_2026_7179_MOESM1_ESM.docx]

**Supplement**

**Table S1**. Definition of general health, developmental, and sociodemographic variables assessed in the school entry examination. Definitions were based on the standardized protocol of the School Entry Health Examination Program (SOPHIA) used in Lower Saxony, Germany.

| Variable | | Definition | Categories |
| --- | --- | --- | --- |
| **Sociodemographic and household characteristics** | | | |
|  | Age | Age at examination | < 6 years; 6 years; 7 years |
|  | Sex | Biological sex | male; female |
|  | Migration background | Based on parental country of birth | none |
|  |  |  | one parent foreign-born |
|  |  |  | both parents foreign-born |
|  | Parental education | Highest educational level of each parent | mother with vocational training |
|  |  |  | mother without vocational training |
|  |  |  | father with vocational training |
|  |  |  | father without vocational training |
|  | Number of siblings | Number of children living in the household | 0; 1; ≥ 2 |
| **Developmental conditions** | | | |
|  | Language problems | Reported or clinically identified delay or impairment in language development | yes; no |
|  |  |  |  |
|  | Delayed walking | Walking independently later than expected developmental milestones | yes; no |
|  |  |  |  |
|  | Early intervention | Participation in early support programs (e.g. speech or developmental therapy) | yes; no |
|  |  |  |  |
|  | Neurological conditions | Neurological or neurodevelopmental conditions identified during examination or reported by parents | yes; no |
|  |  |  |  |
| **Perinatal factors** | | | |
|  | Preterm birth | Birth before 37 completed weeks of gestation | yes; no |
|  | Low birthweight | Birth weight <2,500 g | yes; no |
| **Chronic health conditions** | | | |
|  | Ear, nose, and throat (ENT) conditions | Chronic or recurrent ENT diseases (e.g. otitis media, hearing problems) | yes; no |
|  | Respiratory conditions | Chronic respiratory diseases (e.g. asthma, recurrent bronchitis) | yes; no |
|  | Cardiovascular conditions | Congenital or acquired cardiovascular diseases | yes; no |
|  | Urinary tract conditions | Chronic or recurrent urinary tract disorders | yes; no |
| **Psychosocial and behavioural conditions** | | | |
|  | Psychosocial conditions | Psychosocial burden reported by parents or identified during examination | yes; no |
|  | Behavioural conditions | Behavioural or emotional problems identified during examination or reported by parents | yes; no |
|  | Disability | Functional limitations affecting participation (e.g. physical or cognitive impairment) | yes; no |
| **History of infectious diseases** | | | |
|  | Measles | History of measles infection | yes; no |
|  | Mumps | History of mumps infection | yes; no |
|  | Varicella | History of chickenpox infection | yes; no |
|  | Pertussis | History of pertussis infection | yes; no |
| **Other health indicators** | | | |
|  | Body Mass Index (BMI) category | Based on BMI percentile categories used in the SOPHIA school entry examination protocol and derived from the reference values published by Kromeyer-Hauschild et al. [39]; underweight <3rd percentile, normal weight 3rd–90th percentile, overweight >90th percentile, obesity >97th percentile. | underweight |
|  |  |  | normal weight |
|  |  |  | overweight |
|  |  |  | obesity |
|  | Completion of routine child health check-ups | Participation in scheduled preventive examinations | complete; incomplete |
